# Impact of Frailty on Recurrence After Catheter Ablation for Atrial Fibrillation: A Systematic Review and Meta-analysis

**DOI:** 10.19102/icrm.2026.17076

**Published:** 2026-07-15

**Authors:** Mustafa Bilal Ozbay, Mehmet Esad Demirhan, Serhat Degirmen, Firat Ozcan

**Affiliations:** 1 Department of Medicine, Penn Medicine Princeton Medical Center, Plainsboro, NJ, USA; 2 Department of Cardiology, Montefiore Medical Center, Bronx, NY, USA; 3 Department of Medicine, Metropolitan Hospital Center, New York, NY, USA; 4 Department of Cardiology, Ankara City Hospital, University of Health Sciences, Ankara, Turkey

**Keywords:** Arrhythmia recurrence, atrial fibrillation, catheter ablation, frailty, meta-analysis

## Abstract

Frailty is an increasingly recognized marker of biological vulnerability in patients with atrial fibrillation (AF). Although catheter ablation (CA) is an established rhythm-control strategy, atrial tachyarrhythmia (AT) recurrence remains common, particularly in patients with advanced age and comorbidity burden. The impact of frailty on post-ablation recurrence has not been systematically quantified. We systematically searched MEDLINE, EMBASE, and the Cochrane Library for studies evaluating the association between frailty and AT recurrence following CA for AF. Eligible studies compared frail and non-frail adults undergoing CA and reported post-procedural recurrence. Pooled risk ratios (RRs) with 95% confidence intervals (CIs) were calculated using a random-effects model. Four studies totaling 1096 patients were included, of whom 266 (24.3%) were frail. Across the included studies, the mean age of participants ranged from approximately 61 to 76 years. Frailty was associated with a significantly higher risk of AT recurrence after CA compared with non-frailty (RR, 1.57; 95% CI, 1.26–1.94; *P* < .001; *I*^2^ = 0%). This association remained significant in subgroup analyses limited to studies with ≤12 months of follow-up (RR, 1.46; 95% CI, 1.13–1.89; *P* = .004; *I*^2^ = 0%). Leave-one-out sensitivity analyses confirmed robustness. In conclusion, frailty is independently associated with increased AT recurrence following CA for AF and should be considered in pre-procedural risk stratification.

## Introduction

Atrial fibrillation (AF) is the most common sustained cardiac arrhythmia and is associated with significant morbidity and impaired quality of life.^1,2^ Catheter ablation (CA) is an established rhythm-control strategy for symptomatic AF and is increasingly performed in older patients.^3,4^ However, atrial tachyarrhythmia (AT) recurrence remains a major limitation of CA, particularly among patients with advanced age and multiple comorbidities.^5,6^

Frailty is a clinical syndrome characterized by reduced physiological reserve and increased vulnerability to stressors.^7^ In patients with AF, frailty has been associated with adverse outcomes, including stroke, bleeding, hospitalization, and mortality.^8,9^ Frailty and AF share common pathophysiological pathways, such as chronic inflammation, autonomic dysregulation, and impaired cardiometabolic reserve, which may adversely affect atrial substrate modification and rhythm maintenance following CA.^9,10^

Frailty instruments incorporate overlapping clinical domains such as comorbidity burden, functional status, physical performance such as grip strength and gait speed, unintentional weight loss, exhaustion, cognitive impairment, and social vulnerability.^11^ Despite differences in structure, these tools reflect a shared construct of reduced physiological reserve, which may have important implications for rhythm outcomes following CA for AF.^11,12^

Several observational studies have evaluated the association between frailty and AF recurrence after CA using validated frailty assessment tools.^6,13–15^ While most studies suggest that frailty is associated with a higher risk of post-ablation recurrence,^6,13,15^ one study reported no significant difference in recurrence rates between frail and non-frail patients at 6 months of follow-up.^14^ Additionally, the modest sample sizes of individual studies limit statistical power, making it challenging to draw definitive conclusions.

Therefore, we conducted a systematic review and meta-analysis to evaluate the association between frailty and AT recurrence following CA in patients with AF.

## Methods

This study was designed in accordance with the Preferred Reporting Items for Systematic Reviews and Meta-Analysis (PRISMA) protocol, the Meta-analysis of Observational Studies in Epidemiology (MOOSE) statement, and the PRISMA 2020 checklist.^16,17^ The protocol was prospectively registered in the International Prospective Register of Systematic Reviews (PROSPERO) database with the registration number “CRD420251269292.” This study is a systematic review and meta-analysis of previously published studies. No human subjects were directly involved, and therefore ethical approval was not required.

### Eligibility criteria

Studies that met the following eligibility criteria were included: (1) randomized controlled trials and nonrandomized study designs, including prospective or retrospective cohorts; (2) comparisons of clinical outcomes between frail and non-frail patients undergoing CA for AF; (3) reporting recurrence of AT following CA; and (4) inclusion of adult patients aged ≥50 years with a follow-up duration of at least 3 months. Studies with overlapping patient populations, those published only as conference abstracts, and case reports or case series were excluded.

### Search strategy and data extraction

We systematically searched PubMed, Cochrane, and EMBASE from inception to December 22, 2025, for studies evaluating the association between frailty and AT recurrence following CA for AF. References of eligible papers and systematic reviews were manually searched for additional studies. The complete search strategy for each database can be found in **Box S1**.

Box S1: Full Search Strategy for Each Database

- PubMed
  (“Atrial Fibrillation”[Mesh] OR “atrial fibrillation” OR “AF”) AND (“Catheter Ablation”[MeSH] OR ablation OR “catheter ablation” OR “radiofrequency ablation” OR “cryoballoon ablation” OR “pulmonary vein isolation” OR PVI) AND (frailty OR frail OR “frailty syndrome”)
- EMBASE
  (‘atrial fibrillation’/exp OR ‘atrial fibrillation’ OR ‘af’) AND (‘catheter ablation’/exp OR ‘ablation’ OR ‘catheter ablation’ OR ‘radiofrequency ablation’ OR ‘cryoballoon ablation’ OR ‘pulmonary vein isolation’ OR ‘pvi’) AND (‘frailty’ OR ‘frail’ OR ‘frailty syndrome’)
- Cochrane Library
  (‘atrial fibrillation’ OR ‘af’) AND (‘ablation’ OR ‘catheter ablation’ OR ‘radiofrequency ablation’ OR ‘cryoballoon ablation’ OR ‘pulmonary vein isolation’ OR ‘pvi’) AND (‘frailty’ OR ‘frail’ OR ‘frailty syndrome’)

Two investigators (M.B.O. and S.D.) independently performed the data search and study selection. Disagreements were resolved by author consensus after reviewing the full article and the eligibility criteria with the senior author (F.O.). Three investigators (M.B.O., S.D., and M.E.D.) independently extracted the data of interest from the studies, and all data points were confirmed by the senior author (F.O.).

### Endpoints

Our main outcome of interest was AT recurrence, which was defined as any documented AT, including AF, atrial flutter, or atrial tachycardia, lasting >30 s during post-ablation follow-up.

### Assessment of the risk of bias

For observational studies, we assessed methodological quality using the Risk of Bias in Non-Randomized Studies of Interventions (ROBINS-I) tool.^18^ Risk-of-bias assessments were independently performed by two investigators (M.B.O. and M.E.D.) for all included studies. Any discrepancies were resolved through discussion with the senior author (F.O.).

### Data analysis

We conducted all data analyses following the recommendations outlined by Cochrane.^19^ Binary endpoints were summarized using the Mantel–Haenszel test with a random-effects model risk ratio (RR) and 95% confidence interval (CI). Because adjusted effect estimates were not consistently reported across all included studies, pooled analyses were based primarily on unadjusted RRs derived from study-level event data. We assessed for heterogeneity using Cochran’s *Q* statistic and Higgins and Thompson’s *I*^2^ statistic. *P* values < .10 and *I*^2^ > 50% were considered significant for heterogeneity. Sensitivity analyses were conducted using a leave-one-out approach. Statistical analyses were performed using Review Manager (RevMan) version 5.4 (The Nordic Cochrane Centre, The Cochrane Collaboration, Copenhagen, Denmark) and R software (version 4.5.2; R Foundation for Statistical Computing, Vienna, Austria) using the meta package.

## Results

As detailed in **Figure 1**, our initial search yielded 179 records. After the removal of duplicate reports and screening based on title and/or abstract, we selected 13 studies for full-text review. Four studies met our inclusion criteria,^6,13–15^ encompassing 1096 patients. Two included studies employed a prospective cohort design,^14,15^ while the remaining two were retrospective cohort studies.^6,13^ A total of 266 patients (24.3%) were classified as frail, while 830 patients (75.7%) were classified as non-frail. Follow-up durations ranged from 6 to 25 months. Only one study reported a follow-up duration exceeding 12 months, with a mean follow-up of 25 months.^13^ Further baseline characteristics can be found in **Table 1**.

**Figure 1:**
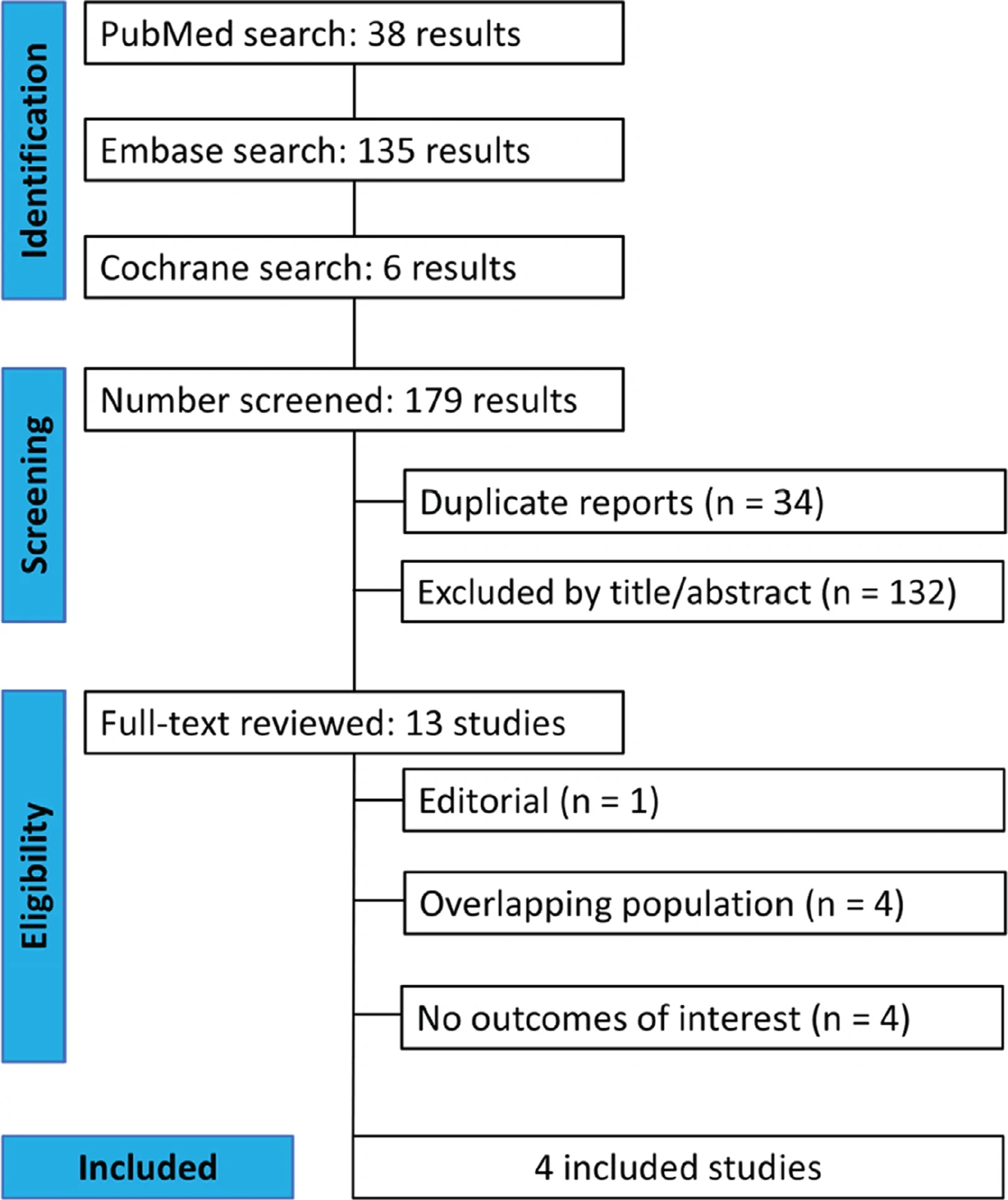
Preferred Reporting Items for Systematic Reviews and Meta-Analyses flow diagram of study screening and selection.

**Table 1:** Study Characteristics

	Xu et al.^6^ (n = 450)		Lin et al.^14^ (n = 195)		Dulai et al.^13^ (n = 248)		Soejima et al.^15^ (n = 203)	
Characteristics	Frail (n = 63)	Not Frail (n = 387)	Frail (n = 47)	Not Frail (n = 148)	Frail (n = 130)	Not Frail (n = 118)	Frail (n = 26)	Not Frail (n = 177)
Study design	Retrospective cohort		Prospective cohort		Retrospective cohort		Prospective cohort	
Year of publication	2025		2025		2023		2023	
Country	China		China		United Kingdom		Japan	
Ablation type	RF		RF		CB		RF or CB	
Evaluation of frailty	mFI-11		FRAIL scale		eFI		FIFSI	
Follow-up duration, months	12		6		25		6	
Age, years^a^	65.8	60.9	72.3	69.8	73.3	72.2	75.5	72.5
Male, %	60.3	69.5	34.0	58.1	44.6	50.0	30.8	66.1
Diabetes mellitus, %	50.8	8.3	38.3	10.1	17.6	6.1	26.9	24.8
Hypertension, %	81.0	45.0	83.0	57.4	58.5	58.3	80.8	71.7
CHF, %	NA	NA	17.0	8.8	17.7	14.8	65.4	36.7
PAF, %	49.2	55.8	48.9	61.5	49.2	57.6	46.2	64.4
Persistent AF, %	50.8	44.2	51.1	38.5	49.2	42.4	42.3	24.8
CHA_2_DS_2_-VASc score, points^a^	2.8	1.4	NA	NA	2.97	2.6	4.0	3.0
CAD, %	20.6	7.0	66.0	31.1	14.6	9.6	NA	NA

*Abbreviations:* AF, atrial fibrillation; CAD, coronary artery disease; CB, cryoballoon; CHF, congestive heart failure; FIFSI, five-item Frailty Screening Index; mFI, modified Frailty Index; NA, not available; PAF, paroxysmal atrial fibrillation; RF, radiofrequency. ^a^Mean or median. *Note:* Rouchen 2025 refers to Xu et al.^6^; Hairong 2025 refers to Lin et al.^14^; Rajdip 2023 refers to Dulai et al.^13^; and Soejima 2023 refers to Soejima et al.^15^.

Xu et al.^6^ and Lin et al.^14^ used radiofrequency (RF) energy for CA, whereas Dulai et al.^13^ employed cryoballoon (CB) ablation. Soejima et al. used either CB or RF ablation; however, their study did not report the proportion of patients treated with each modality.^15^ Frailty assessment varied across studies and was performed using different validated instruments, including the 11-item modified Frailty Index, the FRAIL scale, the electronic Frailty Index, and the five-item Frailty Screening Index **(Tables S1–S4)**. All included studies were published within the past 5 years.

**Table S1:** The Modified Frailty Index

mFI-11 Components
1. Diabetes mellitus
2. Functional dependence (partial or total)
3. Chronic obstructive pulmonary disease or pneumonia
4. Congestive heart failure
5. Myocardial infarction
6. Percutaneous coronary intervention, cardiac surgery, or angina
7. Hypertension requiring medication
8. Peripheral vascular disease or rest pain
9. Impaired sensorium
10. History of transient ischemic attack or cerebrovascular accident
11. Cerebrovascular accident with residual neurologic deficit

The presence of three or more deficits from this table meets the criteria for frailty. *Note:* The mFI is a validated tool derived from the Canadian Study of Health and Aging Frailty Index and is widely used in surgical and cardiovascular outcomes research to quantify patient frailty.^44^

**Table S2:** The FRAIL Scale

FRAIL Components
F—Fatigue: Usually defined as feeling tired *most or all of the time* R—Resistance: Difficulty climbing *one flight of stairs* A—Ambulation: Difficulty walking *one block* (≈100 m) I—Illnesses: Presence of ≥5 chronic illnesses, commonly including hypertension, diabetes, cancer, chronic lung disease, heart disease, stroke, and kidney disease L—Loss of weight: *Unintentional weight loss ≥5%* (or ≥10 lb/4.5 kg) in the past year

*Note:* A patient is considered frail if three or more components from this table are present.^45^

**Table S3:** The Electronic Frailty Index

Frailty Category
≤0.12—Fit 0.13–0.24—Mild frailty 0.25–0.36—Moderate frailty >0.36—Severe frailty

*Note:* The Electronic Frailty Index is a deficit-accumulation frailty measure derived from electronic health records and is calculated as the number of deficits present divided by 36.^46^

**Table S4:** The Five-item Frailty Screening Index (FSI-5)

Components of the FSI-5
Unintentional weight loss: ≥2–3 kg in the past 6 months Low physical activity: Not engaging in regular exercise or physical activity Slow walking speed (self-reported): Feeling slower than before or difficulty walking Exhaustion/fatigue: Feeling tired without reason

The FSI-5 is a brief, self-reported frailty screening tool developed and validated primarily in Asian (especially Japanese) populations.^35^

In the pooled analysis, frail patients exhibited a significantly higher risk of AT recurrence compared with non-frail patients undergoing CA (RR, 1.57; 95% CI, 0.26–1.94; *P* < .0001; *I*^2^ = 0%; **Figure 2**). We also performed a subgroup analysis of patients with a follow-up duration of ≤12 months, which similarly revealed a significantly higher risk of recurrence among frail patients undergoing CA for AF (RR, 1.46; 95% CI, 1.13–1.89; *P* = .004; *I*^2^ = 0%; **Figure 3**).

**Figure 2:**
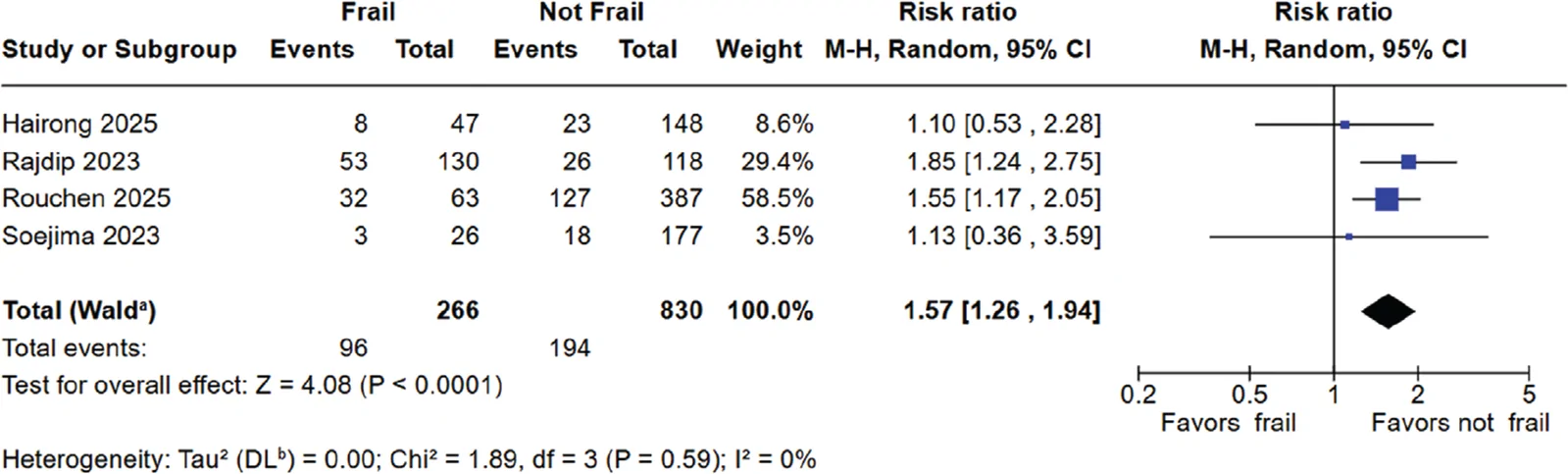
The incidence of atrial tachycardia recurrence was significantly higher in frail patients than in non-frail patients following catheter ablation for atrial fibrillation. *Abbreviations:* CI, confidence interval; DL, DerSimonian–Laird; M–H, Mantel–Haenszel. ^a^Wald test. ^b^DerSimonian–Laird method. *Note:* Rouchen 2025 refers to Xu et al.^6^; Hairong 2025 refers to Lin et al.^14^; Rajdip 2023 refers to Dulai et al.^13^; and Soejima 2023 refers to Soejima et al.^15^.

**Figure 3:**
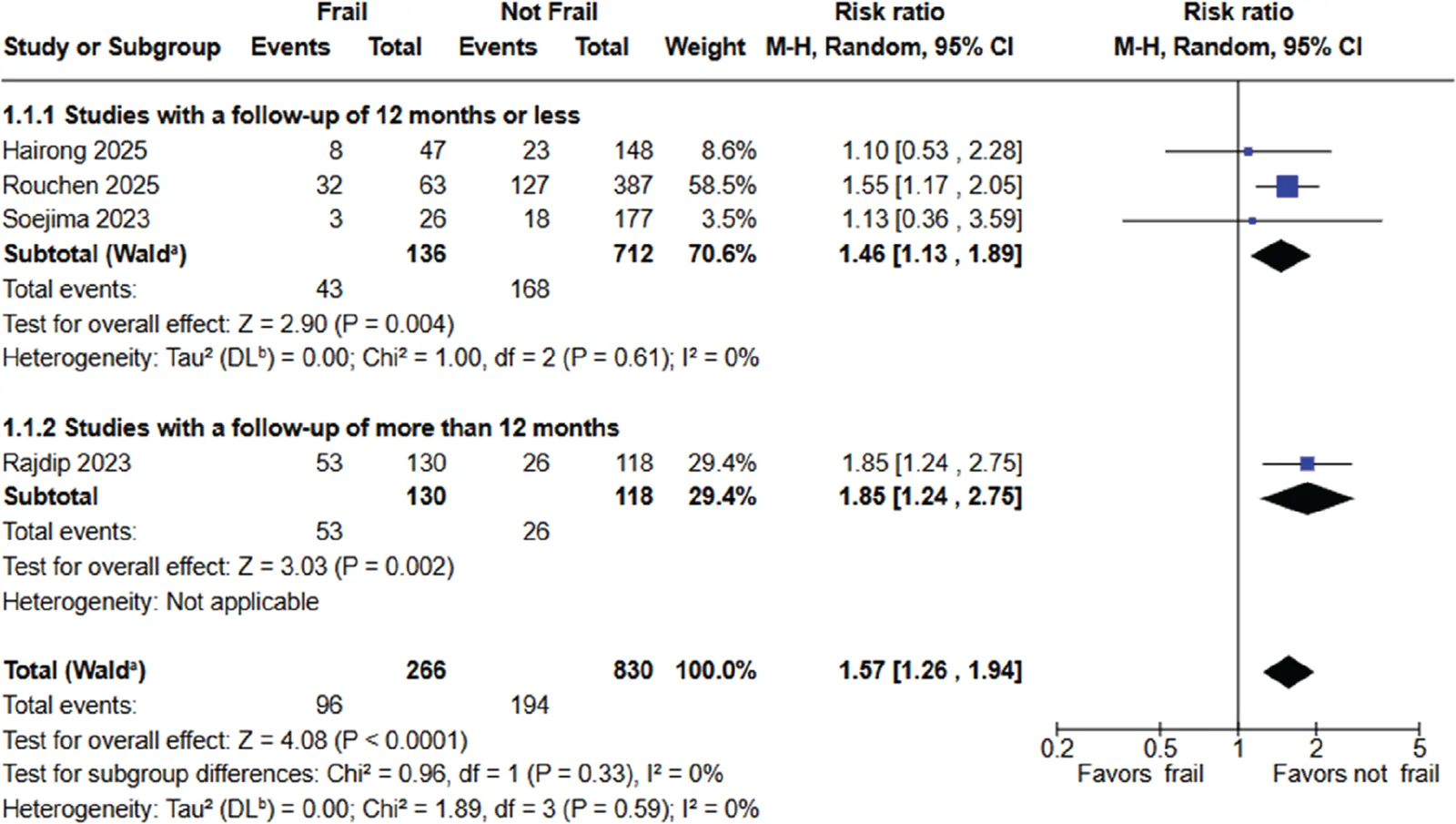
A subgroup analysis of patients with a follow-up of ≤12 months demonstrated a significantly higher risk of recurrence among frail patients following catheter ablation for atrial fibrillation. *Abbreviations:* CI, confidence interval; DL, DerSimonian–Laird; M–H, Mantel–Haenszel. ^a^Wald test. ^b^DerSimonian–Laird method. *Note:* Rouchen 2025 refers to Xu et al.^6^; Hairong 2025 refers to Lin et al.^14^; Rajdip 2023 refers to Dulai et al.^13^; and Soejima 2023 refers to Soejima et al.^15^.

In the subgroup analysis by age category, frailty was associated with a significantly higher risk of the outcome in both studies that exclusively enrolled patients aged ≥65 years (RR, 1.59; 95% CI, 1.14–2.23; *P* = 0.006; *I*^2^ = 0%; **Figure 4**) and studies that included both patients aged ≥65 years and those aged <65 years (RR, 1.55; 95% CI, 1.17–2.05; *P* = 0.002; **Figure 4**). There was no statistically significant difference between subgroups (*P* for subgroup difference = 0.90; **Figure 4**).

**Figure 4:**
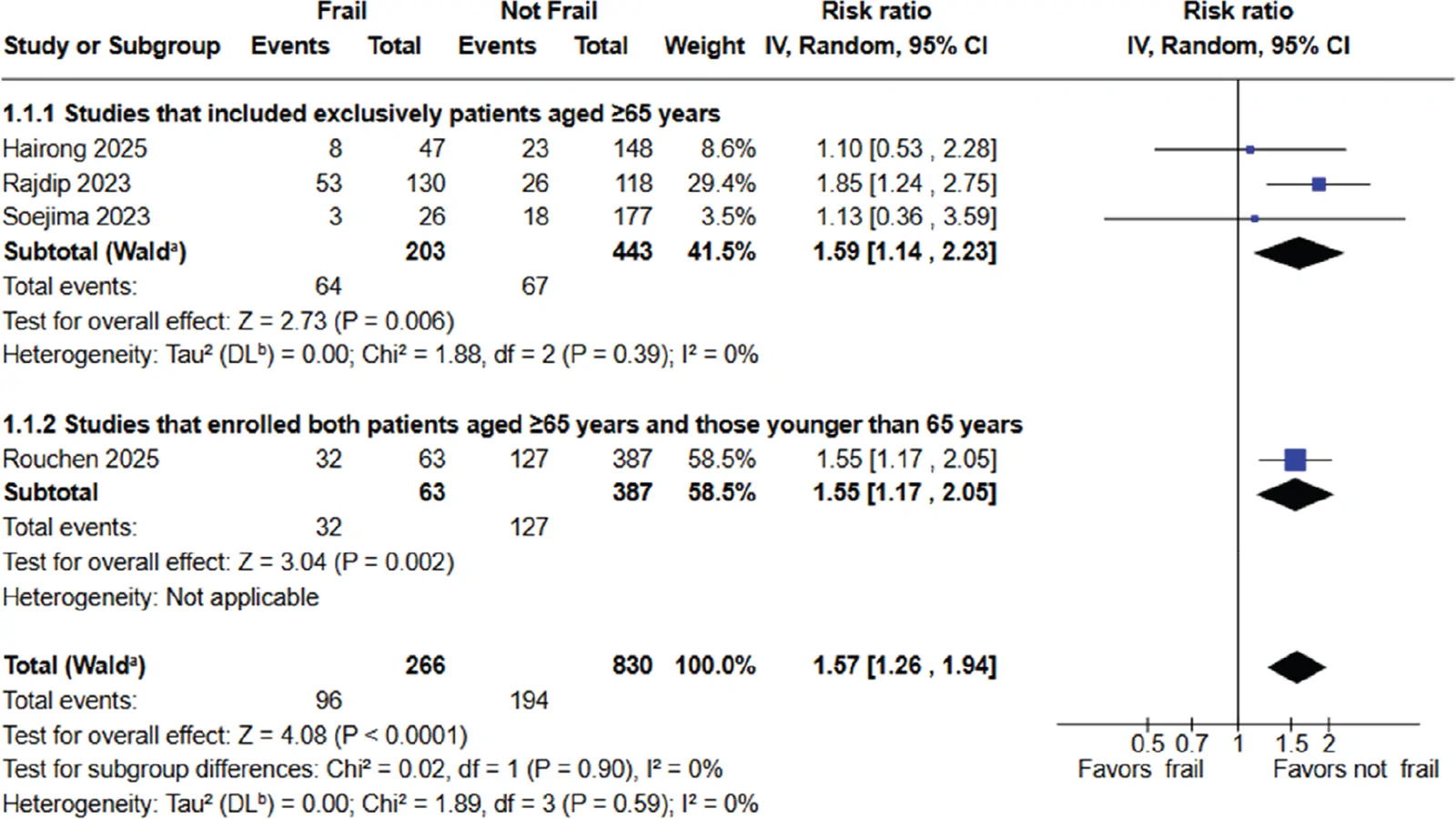
A subgroup analysis of studies including exclusively patients aged ≥65 years demonstrated a significantly higher risk of recurrence among frail patients following catheter ablation for atrial fibrillation. *Abbreviations:* CI, confidence interval; IV, inverse variance. ^a^Wald test. ^b^DerSimonian–Laird method. *Note:* Rouchen 2025 refers to Xu et al.^6^; Hairong 2025 refers to Lin et al.^14^; Rajdip 2023 refers to Dulai et al.^13^; and Soejima 2023 refers to Soejima et al.^15^.

The overall risk of bias across the included studies was rated to be moderate **(Table S5)**. In leave-one-out sensitivity analysis, sequential omission of each study did not significantly alter the direction or magnitude of the association between frailty and recurrence after CA (**Figure S1**). The pooled RR remained statistically significant across all iterations, ranging from 1.46 to 1.62, with consistently low heterogeneity (*I*^2^ = 0%) (**Figure S1**).

**Table S5:** Risk-of-bias Summary for Non-randomized Studies (ROBINS-I)^18^

Study	Bias Due to Confounding	Bias in Selection of Participants	Bias in Classification of Interventions	Bias Due to Deviations from Intended Interventions	Bias Due to Missing Data	Bias in Measurement of Outcomes	Bias in Selection of the Reported Result	Overall Risk-of-bias Judgment
Lin et al.^14^	Moderate	Moderate	Low	Low	Low	Moderate	Low	Moderate
Dulai et al.^13^	Moderate	Moderate	Low	Low	Moderate	Moderate	Low	Moderate
Xu et al.^6^	Moderate	Moderate	Low	Low	Low	Low	Low	Moderate
Soejima et al.^15^	Moderate	Moderate	Moderate	Low	Moderate	Moderate	Low	Moderate

*Abbreviation:* ROBINS-I, Risk of Bias in Non-Randomized Studies of Interventions.

**Figure S1:**
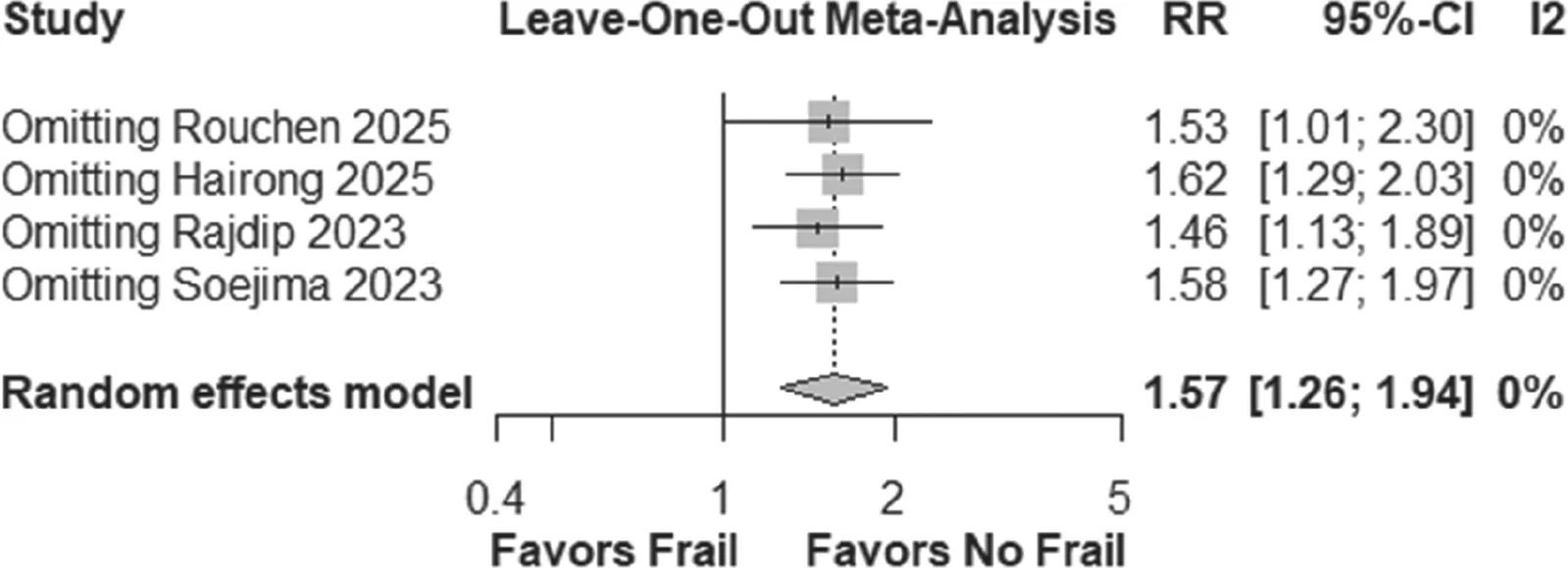
Leave-one-out analysis for recurrence of atrial tachyarrhythmias. *Abbreviations:* CI, confidence interval; RR, risk ratio. *Note:* Rouchen 2025 refers to Xu et al.^6^; Hairong 2025 refers to Lin et al.^14^; Rajdip 2023 refers to Dulai et al.^13^; and Soejima 2023 refers to Soejima et al.^15^.

## Discussion

In this systematic review and meta-analysis of four observational studies^6,13–15^ comprising 1096 patients undergoing CA for AF, we evaluated the association between frailty status and the risk of AT recurrence following CA. The main findings of the pooled analyses were as follows: (1) frail patients exhibited a significantly higher risk of AT recurrence compared with non-frail patients and (2) this association remained consistent in subgroup analyses limited to studies with a follow-up duration of ≤12 months.

Frailty represents a multidimensional state of biological vulnerability that may adversely influence rhythm outcomes after CA through several mechanisms.^20–22^ Frail patients often exhibit a higher burden of systemic inflammation, autonomic imbalance, sarcopenia, and cardiometabolic dysfunction, all of which have been implicated in adverse atrial remodeling and impaired maintenance of sinus rhythm.^10,23,24^ In addition, frailty is frequently accompanied by greater comorbidity burden, including hypertension, diabetes, heart failure (HF), and chronic kidney disease (CKD), which may contribute to a more advanced atrial substrate and reduced efficacy of ablation.^25,26^ These shared pathophysiological pathways provide a plausible biological explanation for the observed association between frailty and post-ablation recurrence.

Several comorbid conditions have been consistently associated with AF recurrence after CA, including HF, hypertension, diabetes mellitus, CKD, and advanced atrial disease.^27–32^ Notably, many of these conditions are highly prevalent among frail individuals and constitute core components of commonly used frailty indices.^33^ In this context, such comorbidities may act as intermediary variables linking frailty to adverse rhythm outcomes, reflecting both structural atrial remodeling and reduced physiological reserve. Frailty, however, extends beyond the mere presence of comorbidities by capturing their cumulative burden, interaction with functional and mobility limitations, and impaired tolerance to procedural stress.^34^ This integrative framework may help explain why frailty remains a clinically meaningful prognostic marker for post-ablation recurrence, even in the presence of established risk factors.

Notably, the association between frailty and recurrence was observed across studies using diverse, validated frailty instruments, including deficit-accumulation (DA) indices^6,13^ and phenotype-based (PB) screening tools.^14,15^ Although frailty was assessed using different validated instruments, each tool captures the shared construct of reduced physiological reserve and vulnerability to stressors.^7,35–38^ From a clinical perspective, the present findings support the incorporation of frailty assessment into the routine evaluation of patients undergoing CA for AF. However, frailty should not necessarily be interpreted as an isolated causal factor. Frailty frequently overlaps with established cardiovascular risk factors such as advanced age, HF, diabetes, and other comorbid conditions, which themselves are known to influence ablation outcomes. Because the studies included in this meta-analysis largely reported unadjusted estimates, it is difficult to fully separate the independent contribution of frailty from the broader burden of multimorbidity. Therefore, frailty may be better conceptualized as an integrative marker of overall physiological vulnerability, capturing the cumulative effects of comorbidities, reduced functional reserve, and systemic health status. In this context, frailty assessment may still provide important prognostic information, as it summarizes multiple interacting risk factors that may contribute to atrial remodeling, impaired recovery, and ultimately higher rates of arrhythmia recurrence following ablation.

Although different frailty instruments were used across studies, they can be broadly classified as DA or PB tools. DA indices rely on routinely documented comorbidities and functional impairments^39^ and may therefore be more practical for electrophysiology practice, whereas PB tools emphasize physical performance and fatigue. DA tools derive frailty scores primarily from comorbidities and clinical deficits documented in the electronic health record, allowing automated or chart-based calculation without additional bedside testing.^6,13^ In contrast, PB tools rely more on patient-reported symptoms or functional measures such as fatigue, mobility, and weight loss.^14,15^ Because of these methodological differences, direct comparison between tools can be challenging. To improve clinical interpretability, we provide a supplementary comparison table **(Table S6)** summarizing the domains, data sources, and practical considerations of each frailty instrument used in the included studies.

**Table S6:** Comparison of Frailty Instruments Used in Included Studies: Domains, Data Source, and Practicality for Electrophysiology Workflow

Tool	Conceptual Model	Main Domains Emphasized	Examples of Items	Data Source/Time Burden	Best-fit Use Case in EP Clinic
mFI-11	DA	*Comorbidity/medical deficits*, functional dependence, neurologic deficits	DM, COPD/pneumonia, CHF, MI, PCI/cardiac surgery/angina, HTN, PVD, impaired sensorium, TIA/CVA, CVA with deficit, functional dependence	Mostly *chart-based*; moderate effort if comorbidities already documented	Pre-ablation *risk stratification using existing EHR data*; research/registry style workflows
eFI (36 deficits)	DA	Broad *multimorbidity + geriatric deficits* across many conditions (EHR-driven)	Calculated as deficits present divided by 36	*EHR-derived*; low incremental burden if automated	*Automated risk flags* in larger systems; population health/pre-procedure risk dashboards
FRAIL scale	PS	*Symptoms + function/mobility*, plus illness burden and weight loss	Fatigue, resistance (stairs), ambulation (1 block), ≥5 illnesses, weight loss	Very quick bedside/self-report	*Rapid EP clinic screening*; pre-op/pre-ablation counseling
FSI-5	PS	Physical performance proxy + nutrition/energy	Weight loss, low activity, slow walking speed (self-report), exhaustion/fatigue	Very quick self-report	*Ultra-brief screening* when clinic flow is tight; may suit settings using short questionnaires

*Abbreviations:* CHF, congestive heart failure; COPD, chronic obstructive pulmonary disease; CVA, cerebrovascular accident; DA, deficit-accumulation; DM, diabetes mellitus; eFI, electronic Frailty Index; EHR, electronic health record; EP, electrophysiology; FSI-5, five-item Frailty Screening Index; HTN, hypertension; mFI, modified Frailty Index; MI, myocardial infarction; PCI, percutaneous coronary intervention; PS, phenotype screening; PVD, peripheral vascular disease; TIA, transient ischemic attack. *Note:* If you want speed at the bedside: FRAIL or FSI-5 (self-report; minimal chart review); if you want comorbidity-driven stratification from documentation: mFI-11 or eFI (chart/EHR-based; scalable when automated).

Despite structural differences, these instruments share core components, such as HF, CKD, diabetes, and functional limitations, that are closely associated with atrial remodeling and reduced rhythm durability, which may explain the consistent association with post-ablation recurrence. However, the absence of patient-level data precludes direct comparison of individual tools or detailed correlation with atrial substrate, underscoring the need for future studies integrating standardized frailty assessment with imaging and electroanatomic data.

One included study did not demonstrate a statistically significant difference in recurrence rates between frail and non-frail patients at 6 months of follow-up.^14^ This finding should be interpreted cautiously, as the study had the smallest sample size among the included cohorts and the shortest follow-up duration, limiting its statistical power to detect clinically meaningful differences.^14^ Importantly, temporal analyses of post-ablation outcomes have consistently shown that divergence in rhythm durability becomes more apparent beyond early follow-up windows, particularly in patients with advanced atrial disease or systemic vulnerability.^40,41^ These findings underscore the importance of adequate follow-up when evaluating ablation outcomes in frail populations.

The clinical implications of these findings are important. Frailty should not be viewed as a contraindication to CA but rather as a prognostic factor that may inform patient selection, counseling, and post-procedural management. Incorporating frailty assessment into pre-ablation evaluation may enhance risk stratification beyond traditional clinical scores and facilitate shared decision-making, particularly in older adults. Furthermore, identification of frailty may allow for targeted interventions, such as optimization of comorbidities, nutritional support, and physical conditioning, which could potentially improve post-ablation outcomes.

Mortality and life expectancy are important considerations when evaluating invasive rhythm-control strategies in frail patients, as frailty has been consistently associated with increased mortality in AF populations independent of age.^9^ However, frailty should not be viewed as a categorical contraindication to CA, but rather as a prognostic framework to support shared decision-making. In patients with advanced frailty and limited life expectancy, treatment goals may reasonably prioritize symptom relief and quality of life over long-term rhythm durability, whereas carefully selected frail patients with meaningful symptom burden and reasonable survival prospects may still derive benefit from ablation. Incorporating frailty assessment into pre-procedural evaluation may therefore help align therapeutic decisions with patient-centered goals rather than exclude patients based solely on age or comorbidity burden.

Several limitations warrant consideration. All included studies were observational, introducing the potential for residual confounding. Definitions of frailty, ablation techniques, and follow-up strategies varied across studies, although statistical heterogeneity was low. The small number of studies and absence of patient-level data limited assessment of interactions between frailty severity, AF subtype, and ablation modality and may not fully reflect the heterogeneity of contemporary clinical practice. Inclusion was restricted to peer-reviewed full-text studies to ensure methodological rigor and reliable outcome ascertainment, which may have excluded unpublished or registry-based data.

An additional consideration relates to the evolution of ablation technologies. The studies included in this meta-analysis primarily evaluated conventional thermal ablation approaches, including RF and CB ablation. However, pulsed field ablation (PFA) has recently emerged as a promising non-thermal ablation modality with several potential advantages, including shorter procedural and ablation times as well as a lower risk of collateral tissue injury and procedural complications due to its tissue-selective mechanism of action.^42,43^ These characteristics may be particularly relevant for frail patients, who often have limited physiological reserve and may be more vulnerable to complications or prolonged anesthesia exposure. In this context, the increasing adoption of PFA could potentially reduce some of the procedural risks associated with CA and expand the feasibility of rhythm-control strategies in frailer patient populations. Nevertheless, data specifically evaluating outcomes of PFA in frail individuals remain limited. Future prospective studies are needed to determine whether the efficiency and safety advantages of PFA translate into improved outcomes in this high-risk group and whether newer ablation technologies may modify the relationship between frailty and post-ablation recurrence.

From a practical perspective, these findings may help inform shared decision-making and patient counseling. Although frailty was associated with a higher risk of arrhythmia recurrence after ablation, it should not be viewed as an absolute contraindication to rhythm-control strategies. Rather, frailty can help set realistic expectations regarding procedural outcomes. For instance, a relative risk of recurrence of approximately 1.5 suggests that frail patients may, on average, experience a shorter duration of sustained sinus rhythm compared with non-frail individuals. Nevertheless, many patients may still derive meaningful benefits from ablation, including symptom relief, a reduction in arrhythmia burden, and improved quality of life. In patients with more advanced frailty or limited life expectancy, discussions may therefore focus on balancing the potential for symptom improvement against the higher likelihood of recurrence and the possibility of repeat interventions. In this setting, frailty assessment may serve as a helpful tool to support individualized counseling and to align treatment decisions with patient goals and overall health status.

Frailty assessment in retrospective studies is also constrained by incomplete documentation of functional domains, such as mobility, which may result in nondifferential misclassification and bias effect estimates toward the null. In addition, frailty indices may be disproportionately influenced by specific comorbidities, precluding separation of their individual effects from the broader frailty construct using study-level data. Nevertheless, frailty instruments are designed to capture the cumulative burden and interaction of comorbidities with functional and physiological vulnerability, potentially providing a more clinically meaningful risk profile than isolated variables.

Finally, this meta-analysis primarily pooled unadjusted study-level estimates, and frailty substantially overlaps with established predictors of recurrence; therefore, frailty should be interpreted as an integrative risk marker rather than a fully independent causal factor. Although frail patients represented a smaller proportion of the pooled population and estimates demonstrated narrow CIs with low heterogeneity, these findings should be interpreted cautiously.

## Conclusion

This meta-analysis demonstrates that frailty is independently associated with an increased risk of AT recurrence following CA for AF, highlighting the prognostic importance of frailty assessment in contemporary electrophysiology practice. Incorporation of frailty evaluation may enhance pre-procedural risk stratification and support shared decision-making. However, the long-term impact of frailty on rhythm durability beyond the first year after ablation remains uncertain, as most included studies had limited follow-up. Future prospective studies with standardized frailty assessment and multivariable-adjusted analyses are needed to better define the independent prognostic role of frailty in AF ablation outcomes.

## Data Availability

All data analyzed in this study are from publicly available published articles. The full dataset used and analyzed during the current study is available from the corresponding author upon reasonable request.
